# Serum Nutritional Biomarkers and All-Cause and Cause-Specific Mortality in U.S. Adults with Metabolic Syndrome: The Results from National Health and Nutrition Examination Survey 2001–2006

**DOI:** 10.3390/nu15030553

**Published:** 2023-01-20

**Authors:** Xinwei Peng, Jingjing Zhu, Henry S. Lynn, Xi Zhang

**Affiliations:** 1 Department of Biostatistics, Key Laboratory on Public Health Safety, School of Public Health, Fudan University, Ministry of Education, Shanghai 200032, China; 2 Clinical Research Unit, Shanghai Ninth People’s Hospital Affiliated to Shanghai Jiao Tong University School of Medicine, Shanghai 200011, China; 3 Clinical Research Unit, Xinhua Hospital, Shanghai Jiao Tong University School of Medicine, Shanghai 200092, China

**Keywords:** biomarkers, carotenoid, potassium, mortality, metabolic syndrome, Bayesian analysis, NHANES

## Abstract

Background: There is limited research on the associations between serum nutritional biomarkers and mortality risk in patients with metabolic syndrome (MetS). Existing studies merely investigated the single-biomarker effect. Thus, this study aimed to investigate the combined effect of nutritional biomarker mixtures and mortality risk using the Bayesian kernel machine regression (BKMR) model in patients with MetS. Methods: We included the MetS patients, defined according to the 2018 Guideline on the Management of Blood Cholesterol from the National Health and Nutrition Examination Survey (NHANES) 2001–2006. A total of 20 serum nutritional biomarkers were measured and evaluated in this study. The Cox proportional hazard model and restricted cubic spline models were used to evaluate the individual linear and non-linear association of 20 nutritional biomarkers with mortality risk. Bayesian kernel machine regression (BKMR) was used to assess the associations between mixture of nutritional biomarkers and mortality risk. Results: A total of 1455 MetS patients had a median age of 50 years (range: 20–85). During a median of 17.1-year follow-up, 453 (24.72%) died: 146 (7.20%) caused by CVD and 87 (5.26%) by cancer. Non-linear and linear analyses indicated that, in total, eight individual biomarkers (α-carotene, β-carotene, bicarbonate, lutein/zeaxanthin, lycopene, potassium, protein, and vitamin A) were significantly associated with all-cause mortality (all *p*-values < 0.05). Results from BKMR showed an association between the low levels of the mixture of nutritional biomarkers and high risk of all-cause mortality with the estimated effects ranging from 0.04 to 0.14 (referent: medians). α-Carotene (PIP = 0.971) and potassium (PIP = 0.796) were the primary contributors to the combined effect of the biomarker mixture. The nutritional mixture levels were found to be negatively associated with the risk of cardiovascular disease (CVD) mortality and positively associated with the risk of cancer mortality. After it was stratified by nutrients, the mixture of vitamins showed a negative association with all-cause and CVD mortality, whereas the mixture of mineral-related biomarkers was positively associated with all-cause and cancer mortality. Conclusion: Our findings support the evidence that nutritional status was associated with long-term health outcomes in MetS patients. It is necessary for MetS patients to be concerned with certain nutritional status (i.e., vitamins and mineral elements).

## 1. Introduction

Metabolic syndrome (MetS) is a series of interrelated metabolic disorders, including abdominal obesity, insulin resistance, dyslipidemia, and elevated blood pressure [1]. Obesity, unhealthy lifestyle such as sedentary behaviors and dietary habits, socioeconomic status, unknown genetic factors, and interactions of these elements are the primary pathogenesis of the MetS [2,3]. In the U.S., the prevalence of MetS is 35% in adults and 50% in people aged 60 years or over [4]. MetS has been recognized as a risk of health outcomes and is significantly related to elevated all-cause mortality [5], especially cardiovascular disease (CVD) mortality, the leading cause of death in MetS patients [6].

Previous evidence has suggested that several nutritional biomarkers were significantly related to health outcomes. Higher serum β-carotene levels were related to lower overall and CVD mortality [7,8]. Serum sodium showed a U-shaped relationship with mortality [9], while both hypernatremia [10] and hyponatremia [11] were risk factors for death. It is reported that serum potassium presented a positive linear association with death in healthy populations [12] and showed a U-shaped [13] relationship with all-cause mortality in patients with chronic disease, such as heart failure, chronic kidney disease, or diabetes mellitus. However, there is a lack of studies focus on the MetS patients, and whether the nutritional status, especially the mixture nutritional status, has an influence on the long-term health outcomes in MetS patients is still uncertain. 

The mechanisms evidence suggested that MetS leading to oxidative stress and inflammation [14,15] might increase the progression of the disease. Increased free fatty acids in obese individuals induce increased production of reactive oxygen species and reactive nitrogen species and subsequent oxidative stress [15,16]. Proinflammatory mediators are upregulated in the insulin-resistant state, promoting malignant transformation and over-proliferation [17]. Therefore, it is plausible that abnormalities of micronutrients with antioxidant properties might be related to MetS and the progression of MetS. For example, serum concentration of 25 hydroxyvitamin D (25(OH)D) was dose-dependently associated with a robust decrement in all-cause and CVD mortality in MetS patients [18,19]. Another view from the perspective of biomarkers and the endocrine system is that vitamin D increment was associated with higher high-density lipoprotein cholesterol (HDL-C) levels and lower serum triglyceride levels [20], which are positive for the development of health outcomes in MetS patients. Additionally, serum lycopene plays a favorable role in MetS because of its antioxidant, anticarcinogenic, and cardioprotective properties [21]. The mortality risk of MetS patients with lower serum lycopene concentrations was significantly higher than those with higher serum lycopene concentrations [14]. However, these investigations mainly evaluated the association of individual nutritional biomarkers with mortality risk [14,22]. In the real-world condition in humans, all nutrients are a mixture and have a healthy effect together [23]; however, still, no study has examined the mixture influence of nutritional status on long-term health outcomes. Hence, we used the data from a representative U.S. population to prospectively evaluate the associations of individual and mixture serum levels of 20 nutritional biomarkers with subsequent mortality risk in patients with MetS.

## 2. Methods

### 2.1. Study Participants

The National Health and Nutrition Examination Survey (NHANES) is a stratified, multi-stage survey among all non-institutionalized populations in the United States. We used data from three continuous cycles between 2001 and 2006, including 31,509 general participants. After excluding the participants younger than 20 years old (*n* = 16,078) and participants without MetS (*n* = 10,824), without follow-up time (*n* = 3), missing values of baseline characteristics (*n* = 812), and missing data on serum biomarker concentrations (*n* = 2337), a total of 1455 MetS patients were identified and included in this study (Figure S1). Ethical approval was given by the NCHS’ Ethics Review Board, and all survey participants provided informed written consent.

### 2.2. Definition of MetS

We identified the MetS according to the 2018 Guideline on the Management of Blood Cholesterol [24]. Participants were defined as MetS if they met any three or above of the following criteria: (1) elevated waist circumference (≥88 cm for women and ≥102 cm for men), (2) elevated triglycerides (≥175 mg/dL) or drug treatment for elevated triglycerides, (3) reduced HDL-C (<50 mg/dL in women and <40 mg/dL in men) or drug treatment for reduced HDL-C, (4) hypertension (systolic ≥ 130 or/and diastolic ≥ 85 mm Hg) or antihypertension drug treatment for a history of hypertension, and (5) elevated fasting glucose (≥100 mg/dL) or drug treatment for elevated fasting glucose.

### 2.3. Laboratory-Based Biomarkers Measurements

This study included a panel of 20 nutritional biomarkers (α-carotene, β-carotene, β-cryptoxanthin, bicarbonate, calcium, chloride, ferritin, folate, iron, lutein/zeaxanthin, lycopene, phosphorus, potassium, protein, sodium, total iron binding capacity, vitamin A, vitamin B12, vitamin D, and vitamin E) available from NHANES 2001–2006. Spot blood specimens were collected by professional phlebotomists at mobile examination centers or at home. Specimens were stored under appropriate frozen temperature (−20 °C) until transfer to National Center for Environmental Health (see details in Appendix A.1 and Appendix A.2).

### 2.4. Ascertainment of Deaths

Participants were matched to the National Death Index unique study identifier to ascertain mortality status through 31 December 2019. Death status was classified using the International Classification of Disease (ICD), the 10th Revision. The primary outcomes included all-cause mortality, cancer mortality, and CVD mortality. We defined death from cancer when C00-C09 was listed as the underlying cause of death. CVD mortality was defined as ICD-10 codes I00–I09, I11, I13, I20–I51, and I60–I69.

### 2.5. Covariates

NHANES used a standardized questionnaire to collect demographic and lifestyle information of participants. We included the variables age, sex, race/ethnicity, body mass index (BMI), smoking status, drinking status, education, physical activities, annual family income, and history of chronic disease. Race/ethnicity was divided into non-Hispanic (NH) white, NH black, Mexican American, and other Hispanic and multi-racial. BMI was calculated as weight in kilograms divided by square of height in meters and classified into three groups: underweight or normal (<25 kg/m^2^), overweight (25–30 kg/m^2^), and obesity (>30 kg/m^2^). Smoking and drinking status were both divided into never, former, and current. Education was categorized as below high school or general educational development, high school graduate, college or Associate of Arts degree, and college graduate or above. Physical activity was based on self-reported exercise intensity over the past month and categorized as low, medium, and high. Annual family income in USD includes 0–19,999, 20,000–34,999, 35,000–75,000, and 75,000 and over. Participants who had been told by doctors that they had coronary heart disease, diabetes, or hypertension were defined as having a history of chronic diseases.

### 2.6. Statistical Analysis

Baseline demographic characteristics and biomarkers are presented as weighted means and standard errors or weighted median and interquartile range (IQR) for continuous variables. Categorical variables were shown as numbers and weighted percentages. We established multivariable-adjusted survey-weighted Cox proportional hazard regression models to estimate the association between each individual biomarker and mortality risk by categorizing each recipient’s biomarker value into quartiles. Using the lowest quartile as the reference, hazard ratios (HRs) and 95% confidence intervals (CIs) for the upper three quartiles were estimated. Additionally, we explored the results of classifying biomarkers concentration into low, normal, and high status (Table S1). We estimated HRs for the low and high statuses with normal status as the reference. Risk factors from previous studies or potential confounders (including age, sex, race/ethnicity, BMI, smoking status, drinking status, education, physical activity, annual family income, and history of chronic disease) were included in the Cox models. Furthermore, we used the three-knots restricted cubic splines model to evaluate the non-linear relationships between individual biomarkers and mortality risk.

The Spearman correlation matrix was used to assess cross-correlations between biomarkers. However, the strong correlations and non-linear and non-additive relationships between biomarkers bring challenges to our study (Figure S2). We used Bayesian kernel machine regression (BKMR) to model the associations between biomarkers and mortality risk [25]. Under a non-parametric Bayesian variable selection framework, BKMR allowed us to examine (1) the posterior mean and 95% CIs of the estimated change in mortality risk when all biomarkers were fixed at a specific percentile compared to when they were fixed at median levels, (2) the relative importance of each biomarker in the model qualified by the posterior inclusion probabilities (PIP), (3) the concentration–response relationship between each biomarker and mortality risk by fixing other biomarkers in the mixture to some given percentiles, and (4) the bivariate concentration–response relationship for biomarkers to explore potential interactions. We further divided 20 biomarkers into three categories: vitamins (α-carotene, β-carotene, β-cryptoxanthin, folate, lutein/zeaxanthin, lycopene, vitamin A, vitamin B12, vitamin D, and vitamin E); mineral-related biomarkers (calcium, ferritin, iron, phosphorus, potassium, sodium, and total iron binding capacity); and others (bicarbonate, protein, and chloride). We used individual Bayesian kernel functions to model within-subgroup combined effects on health outcome, respectively. All BKMR models were adjusted for covariates (age, sex, race/ethnicity, BMI, smoking status, drinking status, education, PA, annual family income, and history of chronic disease) and were fit with 10,000 iterations using an MCMC (Markov chain Monte Carlo) sampler.

The sample weights and complex sampling were considered in all analyses. All analyses were performed by using SAS version 9.4 (SAS Institute, Inc., Cary, NC, USA), and R software version 4.2.1 (R Core Team, Auckland, USA) with “bkmr” package (version 0.2.2) constructed the BKMR model.

## 3. Results

### 3.1. Participants’ Characteristics

Overall, a total of 1455 MetS adults were included; the median age was 50 (range: 20–85) years, 62.30% were females, and 73.06% were NH white (Table 1). Of them, more than half were obese (57.56%), and more than 90% were overweight or obese. Nearly 50% had a college or above degree and annual family income higher than USD 35,000 (55.65%), and physical activity frequency was high (37.55%). More than half of MetS were current or former smokers (51.58%), current drinkers (64.87%), and had a chronic disease history (56.55%). Considering the components of MetS separately, more than 80% of MetS patients had “elevated waist circumference” (85.05%) and “reduced HDL-C” (84.61%); “elevated fasting glucose” occurred the least, accounting for about 51% of all participants. By the end of 2019, a total of 453 (24.72%) died with a median follow-up time of 17.1 years; 87 (5.26%) of these were from cancer, and 146 (7.20%) were from CVD.

### 3.2. Individual Nutritional Status and Mortality Risk

Among 20 nutritional biomarkers, four biomarkers were significantly associated with all-cause mortality after being adjusted for multiple comparisons (Table 2); three biomarkers (α-carotene, β-cryptoxanthin, and chloride) were negatively associated with the risk of all-cause mortality, whereas potassium was positively related to mortality risk. When using the cutoff points based on the previous literature, the results of α-carotene and potassium were consistent with the previous, while β-carotene and lycopene had a significant negative correlation with mortality (Table S2). The restricted cubic spline model indicated that eight biomarkers had a significant association with all-cause mortality (*p* for overall < 0.05) (Figure S3): a U-shaped relationship between concentrations of β-carotene, bicarbonate, protein, and vitamin A and all-cause mortality; an L-shaped or inverted L-shaped relationship between lycopene, potassium, and all-cause mortality; and a negative linear relationship between α-carotene and lutein/zeaxanthin and mortality risk.

### 3.3. Mixture Nutritional Status and Mortality Risk

**Figure 1:**
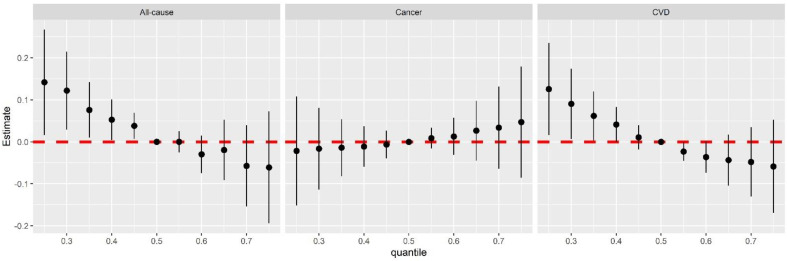
The estimated associations of all biomarker mixtures with all-cause, cancer, and CVD mortality risk with 95% confidence intervals. The X−axis shows percentiles of biomarkers mixtures. The Y−axis shows the estimated change in risk of all−cause mortality. The lines of the plot show the overall effects of the mixture (estimates and 95% CIs) in mortality risk when biomarkers are all at a particular percentile compared to when biomarker are s set at the 50th percentile.

We further investigated the association between the mixture of 20 nutritional biomarkers and mortality risk. Compared with the 50th nutritional mixture, the risk of all-cause mortality was significantly elevated as the mixture concentration decreased, which estimated effects ranging from 0.04 to 0.14 (Figure 1). A weak linear trend was indicated between an elevation of biomarker mixtures and a high risk of cancer mortality but without statistical significance, whereas a significant inverse linear relationship was presented for CVD mortality risk. The PIP values larger than 0.5 are plausibly an important predictor of outcome [26]. The PIPs of α-carotene and potassium were 0.971 and 0.796 for all-cause mortality risk, respectively, suggesting primary contributions from α-carotene and potassium to the mixture association with all-cause mortality. The PIP of α-carotene was 0.690 for CVD mortality risk, and no biomarkers had a dominant impact on the association between the mixture and cancer mortality (all PIPs < 0.5) (Table S3).

**Figure 2:**
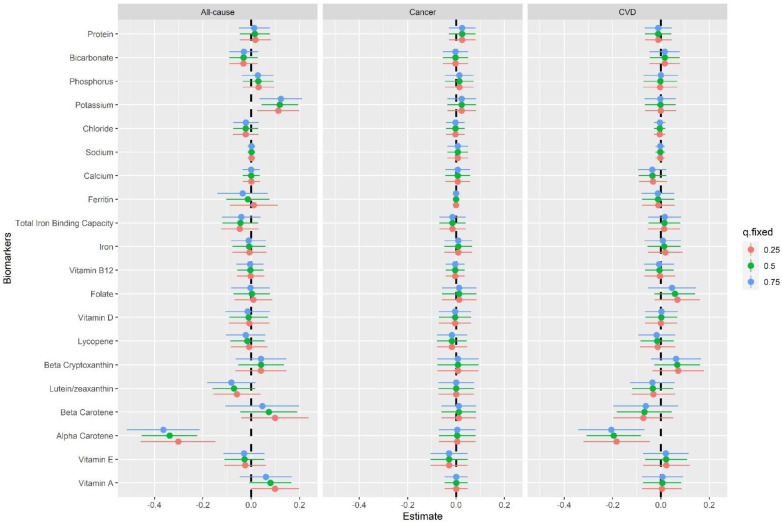
Single−biomarker health effects (95% CI), defined as the change in the response associated with a change in a particular biomarker from its 25th to its 75th percentile, where all of the other biomarkers are fixed at a specific quartile. Red, green, and blue represent the 25th percentile, 50th percentile, and 75th percentile, respectively.

We then examined the single-biomarker healthy influence in the mixture, which was defined as the related change in mortality risk from an IQR concentration increase in a specific biomarker level when other biomarkers in the mixture were fixed at a specific percentile. α-Carotene showed significant negative associations with all-cause and CVD mortality, whereas potassium showed a positive association with all-cause mortality (Figure 2). Specifically, an IQR increase in α-carotene concentration (from 0.026 μmol/L to 0.086 μmol/L) was related to a 0.30 (95% CI: 0.15–0.46), 0.34 (95% CI: 0.22–0.45), and 0.36 (95% CI: 0.21–0.51) SDs decrease in risk of all-cause mortality, respectively, when other biomarkers were fixed at the 25th, 50th, and 75th quantiles. An IQR increase in potassium levels (from 3.8 mmol/L to 4.3 mmol/L) was linked with a 0.11 (95% CI: 0.02–0.20), 0.12 (95% CI: 0.04–0.19), and 0.12 (95% CI: 0.03–0.21) SDs increment in risk of all-cause mortality, respectively. Considering the multiple associations between nutritional biomarkers, we fixed the other nutritional biomarkers at median levels, and the shape of the associations between the specific biomarker and mortality risk was different from the trend presented by individual biomarker analysis (Figure S4). For example, the relationships of bicarbonate, lycopene, and protein with all-cause mortality were no longer U-shaped and became monotonic; and a J-shaped relationship was presented for potassium levels.

**Figure 3:**
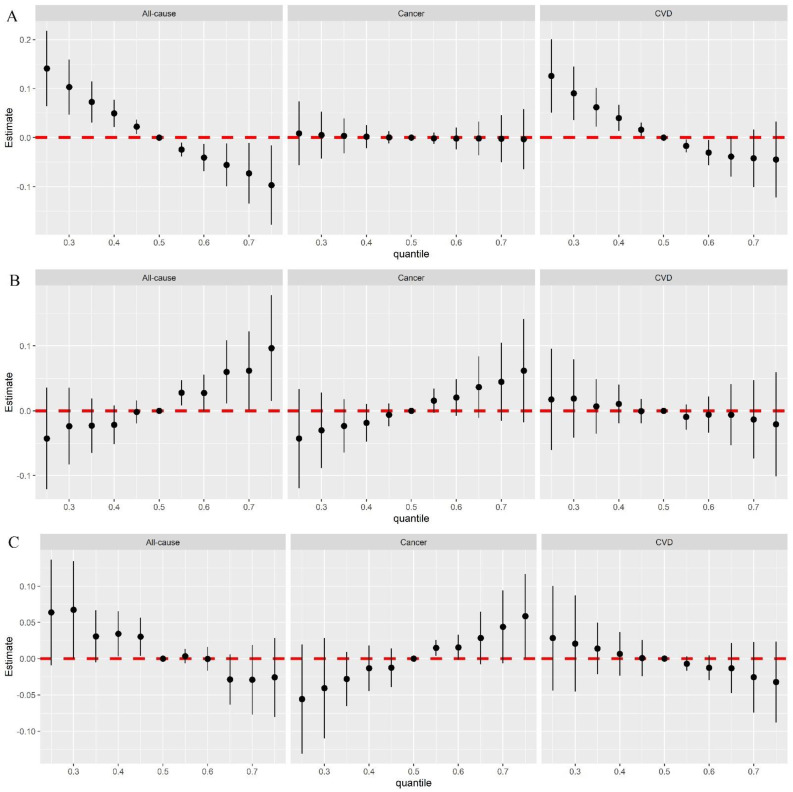
The estimated associations of (**A**) vitamin biomarkers mixture, (**B**) trace metal biomarker mixtures, and (**C**) other biomarker mixtures with all−cause and cause−specific mortality risk with 95% confidence intervals. The X−axis shows percentiles of biomarkers mixtures. The Y−axis shows the estimated change in risk of all−cause mortality. The lines of the plot show the overall effects of the mixture (estimates and 95% CIs) in mortality risk when biomarkers are all at a particular percentile compared to when biomarkers are set at the 50th percentile.

After establishing BKMR models for vitamins, mineral-related biomarkers, and other biomarkers separately, we noted that the elevated concentrations of vitamin mixtures were significantly related to a low mortality risk, especially for CVD mortality (Figure 3A); and an increment in mineral-related biomarker mixtures was linked with high all-cause mortality risk and cancer risk (Figure 3B). The decrement of the other biomarker mixtures indicated a significantly high risk of all-cause mortality (Figure 3C), whereas an inverse relationship for cancer mortality was shown. α-Carotene and potassium dominated the primary influence of vitamin- and mineral-related mixtures on risk of all-cause mortality, respectively (PIP for α-carotene = 0.976, PIP for potassium = 0.815), whereas for risk of CVD mortality, it was α-carotene (PIP = 0.778) (Figures S5 and S6). When the concentrations of other mineral-related biomarkers were fixed at median levels, an IQR increase in bicarbonate (from 22 mmol/L to 25 mmol/L) was linked with a 0.10 (95% CI: 0.03–0.18) SD decrement in risk of all-cause mortality, and an IQR elevated in protein (from 70 g/L to 76 g/L) was related to 0.12 (95% CI: 0.04–0.21) SD increment in risk of cancer mortality (Figure S7).

## 4. Discussion

Our findings provide emerging evidence in support of the associations between low mixture nutritional status and increased risks of all-cause and CVD mortality; further, low mixtures of vitamins and high mixtures of mineral-related biomarkers indicated a high risk of mortality. Additionally, carotene and potassium contributed to the primary influences of mixture nutritional status on mortality risks.

Extensive studies have examined whether nutritional biomarkers are associated with mortality in patients with MetS; however, most of them merely involved single-biomarker exposure [14,18,22]. As we know, in the real world, the human metabolism and immune system always interact and are regulated by the combined effects of the whole nutritional status [23]. In statistical analysis, the highly correlated multiple nutritional biomarkers might be influenced by collinearity, which might lead to a bias estimation by using the traditional regression model [27]. Therefore, in this study, we applied BKMR, which used the Bayesian kernel function, to estimate the combined effects of biomarker mixtures. Moreover, the BKMR model could take the potentially complex relationships between biomarkers into accounts, such as nonlinear relationships and non-additive correlations between biomarkers and mortality risk, as well as identify interactions between biomarkers.

With the help of the BKMR model, we noted a negative association between biomarkers mixtures and all-cause mortality. α-Carotene and potassium were the primary contributing nutritional biomarkers for the association with mortality. Mortality risk tended downward with the elevation of vitamin mixtures but upward for metal element mixtures. These findings are supported by previous evidence [8,28,29,30,31,32,33,34]. The potential underlying mechanism of benefits from α-carotene on mortality risk in MetS patients is mainly due to its regulatory effects on Nrf2 and NF-κB [35,36]. In MetS patients, insulin resistance leads to deterioration into impaired glucose tolerance, which produces oxidative stress, while α-carotene is a natural antioxidant. α-Carotene could interact with and potentiate the Nrf2 pathway and the subsequent activation of the expression of a series of antioxidant and cytoprotective enzymes [37]. Elevated glucose induces NF-κB increase, which promotes the transcription of inflammatory cytokines and causes inflammation. α-Carotene restrained the level and gene expression of NF-κB and attenuated the inflammatory response [38]. In addition, carotenoids play a critical role in modulating adiposity [39] and preventing the dysbiosis of intestinal flora [40]. α-Carotene endorses effects on adipogenesis controlling and glucose/insulin homeostasis, and its biological activities were related to anti-inflammatory response mediation [41], which contributes to the reduction of atherosclerosis and cardiovascular disease risk in MetS patients. Taking all these together, α-carotene might be a predominant protective biomarker for mortality risk in MetS patients.

In the general population, it is reported that abnormal potassium concentrations were related to increment all-cause and CVD mortality risk [42,43,44,45]. Our data also suggested a J-shaped relationship between serum potassium concentrations and mortality risk in MetS patients, which has been reconfirmed by the BKMR model after considering the complex combined association of all these nutritional biomarkers. Potassium homeostasis might partially explain the association in the mechanism. The potassium status affects the resting membrane potential of cardiomyocytes and the activity of potassium channels, all of which are important factors for the cardiac electrical cycle [43]. High serum potassium levels (>5.5 mmol/L) could cause death by induction of acute cardiac arrhythmia [44,46]. MetS individuals, a high-risk population of cardiovascular disease [47], had a higher 10-year cumulative incidence of mortality when the serum potassium levels increased [44]. Serum potassium concentration possibly drove the negative correlation between the nutrient mixture and the risk of death. Another explanation is that in MetS, blood-flow restriction due to obesity produces local acidosis [48]. Metabolic acidosis could induce extracellular potassium increment, which might affect cardiac contractility and lead to adverse health outcome [49]. This means potassium might be a critical nutritional biomarker and play a key effect in health outcomes in MetS patients.

Our findings are based on a study from a large-scale and representative U.S. population. The concentration of biomarkers was provided by laboratory measurement, which is more accurate than memory-based dietary questionnaires. In addition, we applied BKMR, a flexible statistical model considering the complex associations between nutritional biomarkers, to evaluate the individual and combined associations of the nutritional biomarker mixtures with mortality. Some biomarkers associated with mortality risk in previous studies were not significant in this study, and this is possible because the associations of biomarkers were influenced by other biomarkers in the same causal pathway in the pathogenesis. Several limitations should be acknowledged. First, high-correlated biomarker co-status might cause a biased marginal effect [50]. Second, our study did not consider the temporal variability of the concentration–response relationship because the measurement of biomarkers is a single time point, which cannot reflect the long-term nutritional status. Third, most of the biomarker status classification criteria in individual analyses were from healthy populations, which might not be applicable to MetS patients. Fourth, some important biomarkers were not considered in our study due to missing measurement data (such as zinc, copper, selenium, and magnesium). Finally, we cannot exclude the possibility of unmeasured confounding in the associations between nutritional biomarkers and mortality risk.

## 5. Conclusions

Nutritional biomarker mixtures concentration was associated with all-cause and CVD mortality in MetS patients. α-Carotene contributed primarily to vitamin mixtures, and the effect of mineral-related biomarker mixtures was mainly driven by potassium. These findings suggest that paying attention to serum biomarkers concentration deficiency is necessary for MetS patients, and taking vitamins and minerals from a diversified and balanced diet rather than from dietary supplements might be a potential way to improve the health of MetS patients.

## Figures and Tables

**Table 1:** Baseline characteristics of 1314 U.S. adults with metabolic syndrome (MetS) in NHANES 2001–2006.

Characteristics	All	Survival	Death	*p*-Value
	(*n* = 1455)	(*n* = 1002)	(*n* = 453)	
Age (years)	47.51 (39.79–60.87)	45.49 (37.83–53.60)	65.20 (50.89–74.11)	<0.001
Female, n (%)	878 (62.30)	662 (49.38)	216 (12.92)	<0.001
Race/ethnicity, n (%)				0.0148
NH White	775 (73.06)	474 (53.09)	301 (19.97)	
NH Black	271 (10.47)	198 (8.51)	73 (1.96)	
Mexican American	305 (7.08)	250 (6.44)	55 (0.64)	
Others	104 (9.39)	80 (7.23)	24 (2.15)	
BMI, n (%)				<0.001
Underweight or normal	157 (9.60)	85 (5.96)	72 (3.64)	
Overweight	512 (32.84)	319 (23.46)	193 (9.39)	
Obesity	786 (57.56)	598 (45.86)	188 (11.70)	
Education, n (%)				0.0015
Below high school or GED	451 (20.59)	286 (13.81)	165 (6.77)	
High school graduate	374 (28.26)	250 (20.65)	124 (7.62)	
Some college or AA degree	373 (29.36)	279 (23.27)	94 (6.09)	
College graduate or above	257 (21.79)	187 (17.55)	70 (4.24)	
Smoking status, n (%)				<0.001
Nonsmoker	721 (48.42)	545 (39.45)	176 (8.97)	
Former smoker	449 (28.87)	259 (19.26)	190 (9.61)	
Current smoker	285 (22.71)	198 (16.57)	87 (6.14)	
Alcohol use, n (%)				0.6851
Nondrinker	263 (15.91)	180 (12.13)	83 (3.78)	
Former drinker	289 (19.22)	203 (14.05)	86 (5.17)	
Current drinker	903 (64.87)	619 (49.10)	284 (15.77)	
Physical activity, n (%)				0.0025
Low	336 (25.27)	269 (20.99)	67 (4.28)	
Medium	488 (37.18)	325 (27.25)	163 (9.93)	
High	631 (37.55)	408 (27.05)	223 (10.51)	
Annual family income, n (%)				<0.001
USD 0–USD 19999	433 (23.23)	257 (14.82)	176 (8.41)	
USD 20000–USD 34999	356 (21.12)	217 (13.87)	139 (7.25)	
USD 35000–USD 74999	433 (32.80)	335 (26.79)	98 (6.00)	
>USD 75000	233 (22.85)	193 (19.80)	40 (3.05)	
History of chronic disease	862 (56.55)	527 (38.64)	335 (17.92)	<0.001
MetS components, n (%)				
Elevated waist circumference	1226 (85.05)	865 (64.95)	361 (20.10)	0.0299
Elevated triglycerides	907 (61.99)	607 (44.82)	300 (17.16)	0.0178
Reduced HDL-C	1216 (84.61)	849 (65.27)	367 (19.34)	0.0061
Hypertension	1119 (75.61)	716 (53.55)	403 (22.05)	<0.001
Elevated fasting glucose	773 (50.80)	503 (36.00)	270 (14.80)	0.0012

**Table 2:** Associations between baseline nutritional biomarkers levels and all-cause mortality (HRs and 95% CIs).

Nutritional Biomarkers	Mean (SD)	HRs (95% CI)			*p* for Linear Trend ^b^
		Quartile 2 ^a^	Quartile 3	Quartile 4	
*α*-carotene (μmol/L)	0.07 (0.00)	0.87 (0.61–1.25)	0.56 (0.38–0.83)	0.56 (0.40–0.78)	0.0027
*β*-carotene (μmol/L)	0.28 (0.01)	0.60 (0.38–0.95)	0.71 (0.54–0.95)	0.72 (0.48–1.09)	0.5785
*β*-cryptoxanthin (μmol/L)	0.15 (0.00)	0.98 (0.74–1.31)	0.84 (0.66–1.06)	0.69 (0.51–0.93)	0.0278
Bicarbonate (mmol/L)	23.57 (0.15)	0.74 (0.55–1.01)	0.43 (0.32–0.58)	0.70 (0.52–0.93)	0.1495
Calcium (mmol/L)	2.36 (0.00)	1.15 (0.74–1.80)	1.22 (0.79–1.88)	1.23 (0.84–1.80)	0.4289
Chloride (mmol/L)	103.03 (0.17)	0.94 (0.69–1.29)	0.73 (0.57–0.93)	0.72 (0.54–0.96)	0.0474
Ferritin (ug/L)	144.30 (5.65)	0.82 (0.60–1.11)	0.99 (0.75–1.30)	1.04 (0.79–1.38)	0.3354
Folate (nmol/L)	33.35 (1.16)	1.02 (0.71–1.47)	1.09 (0.79–1.51)	1.09 (0.75–1.57)	0.774
Iron (μmol/L)	14.65 (0.24)	0.76 (0.53–1.09)	0.83 (0.54–1.26)	0.95 (0.74–1.21)	0.774
Lutein/zeaxanthin (μmol/L)	0.26 (0.01)	0.70 (0.54–0.92)	0.55 (0.39–0.78)	0.63 (0.43–0.93)	0.1276
Lycopene (μmol/L)	0.40 (0.01)	0.75 (0.61–0.94)	0.70 (0.52–0.94)	0.88 (0.67–1.15)	0.5785
Phosphorus (mmol/L)	1.20 (0.01)	1.36 (1.05–1.76)	1.28 (0.75–2.19)	1.39 (1.05–1.83)	0.2414
Potassium (mmol/L)	4.05 (0.02)	1.05 (0.67–1.65)	0.99 (0.76–1.30)	1.64 (1.13–2.38)	0.0474
Protein (g/L)	72.59 (0.22)	0.76 (0.56–1.04)	0.92 (0.65–1.31)	1.16 (0.80–1.68)	0.3823
Sodium (mmol/L)	138.61 (0.14)	0.80 (0.54–1.17)	0.91 (0.67–1.24)	0.87 (0.61–1.23)	0.6788
Total iron binding capacity (μmol/L)	65.19 (0.46)	0.93 (0.63–1.37)	0.96 (0.67–1.37)	0.87 (0.54–1.41)	0.7293
Vitamin A (μmol/L)	2.22 (0.03)	0.91 (0.70–1.18)	1.42 (0.95–2.11)	1.25 (0.91–1.71)	0.1495
Vitamin B12 (pmol/L)	373.38 (10.29)	1.07 (0.71–1.62)	0.98 (0.58–1.66)	1.01 (0.72–1.42)	0.9411
Vitamin D (nmol/L)	58.35 (1.07)	1.01 (0.61–1.69)	0.98 (0.61–1.56)	0.99 (0.66–1.49)	0.9411
Vitamin E (μmol/L)	34.37 (0.50)	1.15 (0.78–1.69)	1.14 (0.82–1.57)	0.88 (0.63–1.24)	0.3354

^a^. The lowest quartiles were used as the reference groups. ^b^. Considering multiple comparisons, the *p*-value was adjusted by the BH method.

## Data Availability

The datasets generated and analyzed during the current study are available on the NHANES website: https://www.cdc.gov/nchs/nhanes/index.htm (accessed on 30 October 2022).

## Supplementary Materials

The following supporting information can be downloaded at: https://www.mdpi.com/article/10.3390/nu15030553/s1, Figure S1: Flowchart of participants included and excluded; Figure S2: Spearman correlation matrix between 20 biomarkers; Figure S3: Non-linear associations between single biomarker and all-cause mortality; Figure S4: Univariate concentration-response functions between 20 biomarker mixtures and all-cause mortality; Figure S5: Single-biomarker health effects (95% CI) in vitamin mixture; Figure S6: Single-biomarker health effects (95% CI) in trace metal element mixture; Figure S7: Single-biomarker health effects (95% CI) in other biomarker mixture; Table S1: Nutritional biomarker status categorization; Table S2: Associations between nutritional biomarkers status and all-cause mortality; Table S3. PIP summary of 20 nutritional biomarkers. [51,52,53,54,55,56,57,58,59,60,61,62,63,64,65,66,67,68,69,70].

## Abbreviations

MetS, metabolic syndrome; BKMR, Bayesian kernel machine regression; NHANES, National Health and Nutrition Examination Survey; CVD, cardiovascular disease; HDL-C, high-density lipoprotein cholesterol; ICD, international classification of disease; BMI, body mass index; HR, hazard ratio; CI, confidence interval; PIP, posterior inclusion probability; IQR, interquartile range.

## Appendix A

### Appendix A.1. Detailed Descriptions of Laboratory Methodology of Serum Biomarkers

1. Alpha-carotene, beta-carotene, beta-cryptoxanthin, lutein/zeaxanthin, lycopene, vitamin A, and vitamin E were measured using high performance liquid chromatography with photodiode array detection. A small volume (100 μL) of serum is mixed with an ethanol solution containing two internal standards- retinyl butyrate and nonapreno-β-carotene (C45). The micronutrients are extracted from the aqueous phase into hexane and dried under vacuum. The extract is redissolved in ethanol and acetonitrile and is filtered to remove any insoluble material. An aliquot of the filtrate is injected onto a C18 reversed phase column and isocratically eluted with a mobile phase consisting of equal parts of ethanol and acetonitrile. Absorbance of these substances in solution is linearly proportional to concentration, thus spectrophotometric methods are used for quantitative analysis. Three wavelengths, approximately corresponding to absorption maxima, namely, 300, 325, and 450 nm, are simultaneously monitored and chromatograms are recorded. Quantitation is accomplished by comparing the peak height of the analyte in the unknown with the peak height of a known amount of the same analyte in a calibrator solution. Calculations are corrected based on the peak height of the internal standard in the unknown compared with the peak height of the internal standard in the calibrator solution. Retinol and the retinyl esters are compared with retinyl butyrate at 325 nm, α-and γ-tocopherol are compared with retinyl butyrate at 300 nm, and the carotenoids are compared with C45 at 450 nm.

2. Bicarbonate measurement:

The LX20 system uses indirect (or diluted) ISE methodology to measure the total CO_2_ level in serum. The system measures the rate of pH change as CO_2_ ions diffuse across a membrane.

3. Calcium measurement:

The LX20 system uses indirect (or diluted) ISE methodology to measure calcium concentration in serum. The system determines calcium concentration by measuring calcium ion activity in solution.

4. Ferritin was measured by using the Bio-Rad Laboratories’ “QuantImune Ferritin IRMA” kit (Bio-Rad Laboratories, 1986), which is a single-incubation two-site immunoradiometric assay (IRMA) based on the general principles of assays as described by Addison et al. (1972) and Miles (1977) and modified by Jeong et al. In this IRMA, which measures the most basic isoferritin, the highly purified 125I-labeled antibody to ferritin is the tracer, and the ferritin antibodies are immobilized on polyacrylamide beads as the solid phase. Serum or ferritin standards (made from human liver) are mixed with the combined tracer/solid-phase antibody reagent, and the mixture is incubated. During incubation, both the immobilized and the 125Ilabeled antibodies bind to the ferritin antigen in the serum or standards, thus creating a “sandwich.”

After incubation, the beads are diluted with saline, centrifuged, and decanted. The level of 125I-labeled ferritin found in the pellets is measured by using a gamma counter. There is a direct relationship between the radioactive levels of the pellets and the amount of endogenous ferritin in the serum or standards, rather than the inverse relationship measured by most radioimmunoassays (RIAs).

5. Serum folate and vitamin B12 are measured by using the Bio-Rad Laboratories “Quantaphase II Folate/vitamin B12” radioassay kit (Bio-Rad Laboratories, 1993). The assay is performed by combining serum or a whole blood hemolysate sample with 125I-folate and 57Co-vitamin B12 in a solution containing dithiothreitol (DTT) and cyanide. The mixture is boiled to inactivate endogenous folate-binding proteins and to convert the various forms of vitamin B12 to cyanocobalamin. The reduced folate and its analogs are stabilized by DTT during the heating. The mixture is cooled and then combined with immobilized affinity-purified porcine intrinsic factor and folate-binding proteins. The addition of these substances adjusts and buffers the pH of the reaction mixture to 9.2. The reaction mixture is then incubated for 1 hour at room temperature.

During incubation, the endogenous and labeled folate and B12 compete for the limited number of binding sites based on their relative concentrations. The reaction mixtures are then centrifuged and decanted. Labeled and unlabeled folate and vitamin B12, binding to immobilized binding proteins, are concentrated in the bottom of the tube in the form of a pellet. The unbound folate and B12 in the supernatant are discarded, and the radioactivity associated with the pellet is counted. Standard curves are prepared by using the pre-calibrated folate/B12 standards in a human serum albumin base. The concentration of the folate and vitamin B12 in the participant’s serum or folate in a participant’s whole blood is calculated from the standard curve.

6. Serum iron and TIBC tests were measured by a modification of the automated AAII-25 colorimetric method, which was based on the procedures of Giovaniello et al. and Ramsey. The method was modified to be performed on an Alpkem Flow Solutions 3000 (rapid-flow analysis) system. Iron was quantitated by measuring the intensity of the violet complex formed in the reaction between ferrozine and Fe++ in acetate buffer and measured at 562 nm. Thiourea was added to complex Cu++, which can also bind with ferrozine and yield falsely elevated iron values. For the TIBC test, serum was mixed with 400 g/dL iron solution to saturate the iron-binding sites of the serum transferrin molecules. Magnesium carbonate was used to remove excess iron. Centrifugation was used to precipitate the magnesium carbonate, and the supernatant was measured for iron content.

7. Inorganic phosphorus reacts with ammonium molybdate in an acidic solution to form ammonium phosphomolybdate with a formula of (NH4) 3 [PO4 (MoO3)12]. The ammonium phosphomolybdate is quantified in the ultraviolet range (340 nm), utilizing a sample blanked endpoint method.

8. Serum total protein measurement: Colorimetric assay

- Sample and addition of R1 (blank reagent)

- Addition of R2 (biuret reagent) and start of the reaction: Divalent copper reacts in alkaline solution with protein peptide bonds to form the characteristic purple-colored biuret complex. Sodium potassium tartrate prevents the precipitation of copper hydroxide and potassium iodide prevents autoreduction of copper. The color intensity is directly proportional to the protein concentration that can be determined photometrically.

9. Serum sodium, potassium, and chloride measurements:

An Ion-Selective Electrode (ISE) makes use of the unique properties of certain membrane materials to develop an electrical potential (electromotive force, EMF) for the measurements of ions in solution.

10. Vitamin D measurement:

NHANES 2001-2006 have been converted by using regression to equivalent 25(OH)D measurements from a standardized liquid chromatography-tandem mass spectrometry (LC-MS/MS) method.

### Appendix A.2. Laboratory Quality Assurance and Monitoring of Serum Biomarkers

Serum specimens are processed, stored, and shipped to the Division of Environmental Health Laboratory Sciences, National Center for Environmental Health, Centers for Disease Control and Prevention for analysis.

The NHANES quality control and quality assurance protocols (QA/QC) meet the 1988 Clinical Laboratory Improvement Act mandates. Detailed quality control and quality assurance instructions are discussed in the NHANES Laboratory/Medical Technologists Procedures Manual (LPM). Vials are stored under appropriate frozen (–20 °C) conditions until they are shipped to National Center for Environmental Health for testing.
