# Supplementary material for: Serum Nutritional Biomarkers and All-Cause and Cause-Specific Mortality in U.S. Adults with Metabolic Syndrome: The Results from National Health and Nutrition Examination Survey 2001–2006
Source: Nutrients. 2023 Jan 20;15(3):553. doi: 10.3390/nu15030553 (PMC9918903; doi:10.3390/nu15030553)
Supplement: Supplementary file 1 [file nutrients-15-00553-s001.zip › nutrients-2081560-supplementary.pdf]

Supplementary Material for "Serum nutritional biomarkers and all-cause and cause-specific mortality in U.S. adults with metabolic syndrome: the results from National Health and Nutrition Examination Survey 2001–2006".

Table of contents

Supplemental Table S1. Nutritional biomarker status categorization.

Supplemental Table S2. Associations between nutritional biomarkers status and all-cause mortality

Supplemental Table S3. PIP summary of 20 nutritional biomarkers.

Supplemental Figure S1. Flowchart of participants included and excluded.

Supplemental Figure S2. Spearman correlation matrix between 20 biomarkers.

Supplemental Figure S3. Non-linear associations between single biomarker and all-cause mortality.

Supplemental Figure S4. Univariate concentration-response functions between 20 biomarker mixtures and all-cause mortality

Supplemental Figure S5. Single-biomarker health effects (95% CI) in vitamin mixture.

Supplemental Figure S6. Single-biomarker health effects (95% CI) in trace metal element mixture.

Supplemental Figure S7. Single-biomarker health effects (95% CI) in other biomarker mixture.

Supplemental Table S1. Nutritional biomarker status categorization.

Biomarkers	Low status cutoff point	High status cutoff point	Reference
α-carotene (μmol/L) [1]	0.032	0.069	
β-carotene (μmol/L)	0.060	1.700	[1]
β-cryptoxanthin (μmol/L) [1]	0.096	0.176	
Bicarbonate (mmol/L)	21	32	[2]
Calcium (mmol/L)	2.130	2.630	[3-5]
Chloride (mmol/L)	96	106	[6]
Ferritin (ug/L) [2]	24/11	336/307	[7, 8]
Folate (nmol/L)	16	109	[9]
Iron (μmol/L)	13	27	[10]
Lutein/zeaxanthin (μmol/L)	0.139	0.487	[11, 12]
Lycopene (μmol/L)	0.220	1.168	[13]
Phosphorus (mmol/L)	0.800	1.450	[14]
Potassium (mmol/L)	3.5	5.5	[15]
Protein (g/L)	60	80	[16]
Sodium (mmol/L)	135	145	[17]
TIBC (μmol/L)	45	72	[10]
Vitamin A (μmol/L)	1.130	2.720	[18]
Vitamin B12 (pmol/L)	145	637	[19]
Vitamin D (nmol/L)	50	75	[20, 21]
Vitamin E (μmol/L)	12.77	39.47	[22]

1. Use tertile as cutoff points.

2. Male/female have different cutoff points.

Reference

1. *Carotene, Beta*. [cited 2022 12.30]; Available from: https://www.mayocliniclabs.com/test-catalog/overview/75178#Clinical-and-Interpretive.
2. Kanda, E., et al., *High serum bicarbonate level within the normal range prevents the progression of chronic kidney disease in elderly chronic kidney disease patients.* BMC Nephrol, 2013. **14**: p. 4.
3. Walker, H.K., W.D. Hall, and J.W. Hurst, *Clinical methods: the history, physical, and laboratory examinations.* 1990.
4. Cooper, M.S. and N.J. Gittoes, *Diagnosis and management of hypocalcaemia.* Bmj, 2008. **336**(7656): p. 1298-302.
5. Frølich, A., *Prevalence of hypercalcaemia in normal and in hospital populations.* Dan Med Bull, 1998. **45**(4): p. 436-9.
6. Morrison, G., *Serum Chloride*, in *Clinical Methods: The History, Physical, and Laboratory Examinations. 3rd edition*, H.K. Walker, W.D. Hall, and J.W. Hurst, Editors. 1990, Butterworths: Boston.
7. Richard, M. and P. Matthew, *Henry's Clinical Diagnosis and Management by Laboratory Methods. 21st ed*. 2007: Elsevier Saunders. 506.
8. Cappellinin, M.D., S.F. Lo, and D.W. Swickels, *Tietz Textbook of Clinical Chemistry and Molecular Diagnostics. 6th ed*, ed. N. Rafai, A.R. Horvath, and C.T. Wittwer. 2018: Elsevier Saunders. 719-775.
9. Pfeiffer, C.M., et al., *Folate status and concentrations of serum folate forms in the US population: National Health and Nutrition Examination Survey 2011-2.* Br J Nutr, 2015. **113**(12): p. 1965-77.
10. Williams, D., A. Kenyon, and D. Adamson, *Chapter Ten - Physiology*, in *Basic Science in Obstetrics and Gynaecology (Fourth Edition)*, P. Bennett and C. Williamson, Editors. 2010, Churchill Livingstone. p. 173-230.
11. Rock, C.L., et al., *Diet and lifestyle correlates of lutein in the blood and diet.* J Nutr, 2002. **132**(3): p. 525s-530s.
12. Schupp, C., et al., *Lutein, zeaxanthin, macular pigment, and visual function in adult cystic fibrosis patients.* Am J Clin Nutr, 2004. **79**(6): p. 1045-52.
13. Cámara, M., et al., *Chapter 11 - Lycopene: A Review of Chemical and Biological Activity Related to Beneficial Health Effects*, in *Studies in Natural Products Chemistry*, R. Atta ur, Editor. 2013, Elsevier. p. 383-426.
14. Koumakis, E., et al., *The Causes of Hypo- and Hyperphosphatemia in Humans.* Calcif Tissue Int, 2021. **108**(1): p. 41-73.
15. Jin, A., et al., *Normal range of serum potassium, prevalence of dyskalaemia and associated factors in Chinese older adults: a cross-sectional study.* BMJ Open, 2020. **10**(10): p. e039472.
16. Busher, J.T., *Clinical Methods: The History, Physical, and Laboratory Examinations. 3rd edition*, H.K. Walker, W.D. Hall, and J.W. Hurst, Editors. 1990, Boston: Butterworths.
17. McKee, M., et al., *'Normal' serum sodium concentration among inpatients over 65 admitted to hospital: an observational study.* Postgrad Med J, 2016. **92**(1083): p. 21-6.
18. Ball, G.F.M., *Vitamins: Their role in the human body*. 2004: Blackwell Publishing. 133-187.
19. Norasyikin, A.W., et al., *Autoimmune polyglandular syndrome presenting with jaundice and thrombocytopenia.* Med Princ Pract, 2014. **23**(4): p. 387-9.
20. Vieth, R., *What is the optimal vitamin D status for health?* Prog Biophys Mol Biol, 2006. **92**(1): p. 26-32.
21. Holick, M.F., et al., *Evaluation, treatment, and prevention of vitamin D deficiency: an Endocrine Society clinical practice guideline.* J Clin Endocrinol Metab, 2011. **96**(7): p. 1911-30.
22. Ball, G.F.M., *Vitamins: Their role in the human body*. 2004: Blackwell Publishing. 234-255.

Supplemental Table S2. Associations between baseline nutritional biomarkers status and all-cause mortality (HRs and 95% CIs).

Nutritional biomarkers	HRs (95% CI)			*p-value* [c]
	Low [a]	Normal [b]	High	
α-carotene (μmol/L)	1.21 (0.90–1.64)	1	0.74 (0.60–0.91)	0.0100
β-carotene (μmol/L)	2.47 (1.55–3.94)	1	0.93 (0.34–2.53)	0.0040
β-cryptoxanthin (μmol/L)	1.02 (0.73–1.42)	1	0.76 (0.57–1.01)	0.0544
Bicarbonate (mmol/L)	1.61 (1.03–2.51)	1	-	0.1169
Calcium (mmol/L)	1.09 (0.44–2.74)	1	0.99 (0.57–1.72)	0.9800
Chloride (mmol/L)	1.12 (0.57–2.24)	1	0.81 (0.60–1.10)	0.5717
Ferritin (ug/L)	0.72 (0.42–1.24)	1	1.16 (0.85–1.59)	0.3574
Folate (nmol/L)	1.18 (0.73–1.89)	1	1.04 (0.49–2.21)	0.8143
Iron (μmol/L)	0.90 (0.72–1.13)	1	0.94 (0.56–1.59)	0.7368
Lutein/zeaxanthin (μmol/L)	1.53 (1.00–2.33)	1	0.95 (0.58–1.56)	0.2384
Lycopene (μmol/L)	1.40 (1.12–1.74)	1	0.00 (0.00–0.00)	<0.0001
Phosphorus (mmol/L)	0.17 (0.02–1.70)	1	1.50 (0.86–2.61)	0.1230
Potassium (mmol/L)	1.02 (0.78–1.33)	1	8.61(2.49-29.80)	0.0200
Protein (g/L)	1.42(0.11-18.53)	1	1.21 (0.90–1.61)	0.5793
Sodium (mmol/L)	1.03 (0.54–1.96)	1	0.71 (0.38–1.31)	0.6576
Total iron binding capacity	1.55 (0.70–3.43)	1	0.86 (0.64–1.16)	0.2718
Vitamin A (μmol/L)	1.94 (0.96–3.94)	1	1.10 (0.90–1.35)	0.1169
Vitamin B12 (pmol/L)	0.60 (0.34–1.04)	1	1.31 (0.85–2.01)	0.2384
Vitamin D (nmol/L)	1.04 (0.74–1.45)	1	1.02 (0.71–1.47)	0.9800
Vitamin E (μmol/L)	2.57 (0.69–9.66)	1	0.78 (0.61–1.00)	0.1464

a. Low, normal, and high-status cutoff points refer to Table S1.
b. The normal groups were used as the reference groups.
c. Considering multiple comparisons, the P-values were adjusted by the BH method.

Supplemental Table S3. PIP summary of 20 nutritional biomarkers.

Biomarkers	All-cause	Cancer	CVD
α-carotene	0.971	0.008	0.690
β-carotene	0.485	0.005	0.393
β-cryptoxanthin	0.127	0.063	0.182
Bicarbonate	0.327	0.142	0.222
Calcium	0.272	0.146	0.401
Chloride	0.398	0.229	0.261
Ferritin	0.112	0.000	0.007
Folate	0.040	0.040	0.429
Iron	0.090	0.037	0.234
Lutein	0.197	0.031	0.178
Lycopene	0.115	0.022	0.126
Phosphorus	0.283	0.115	0.201
Potassium	0.796	0.162	0.272
Protein	0.264	0.259	0.280
Sodium	0.291	0.214	0.321
TIBC	0.342	0.093	0.234
Vitamin A	0.476	0.007	0.252
Vitamin B12	0.025	0.006	0.093
Vitamin D	0.138	0.033	0.047
Vitamin E	0.107	0.034	0.233

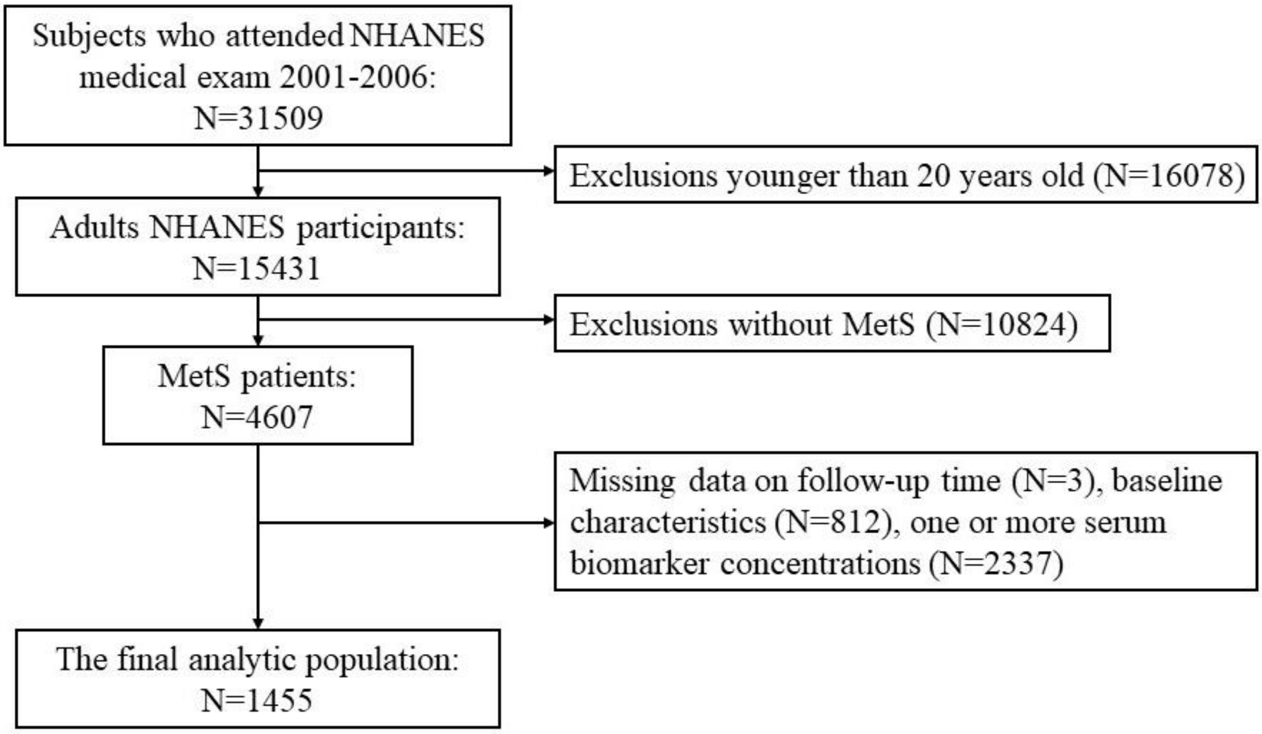

Supplemental Figure S1. Flowchart of participants included and excluded in the final analysis (N=1455).

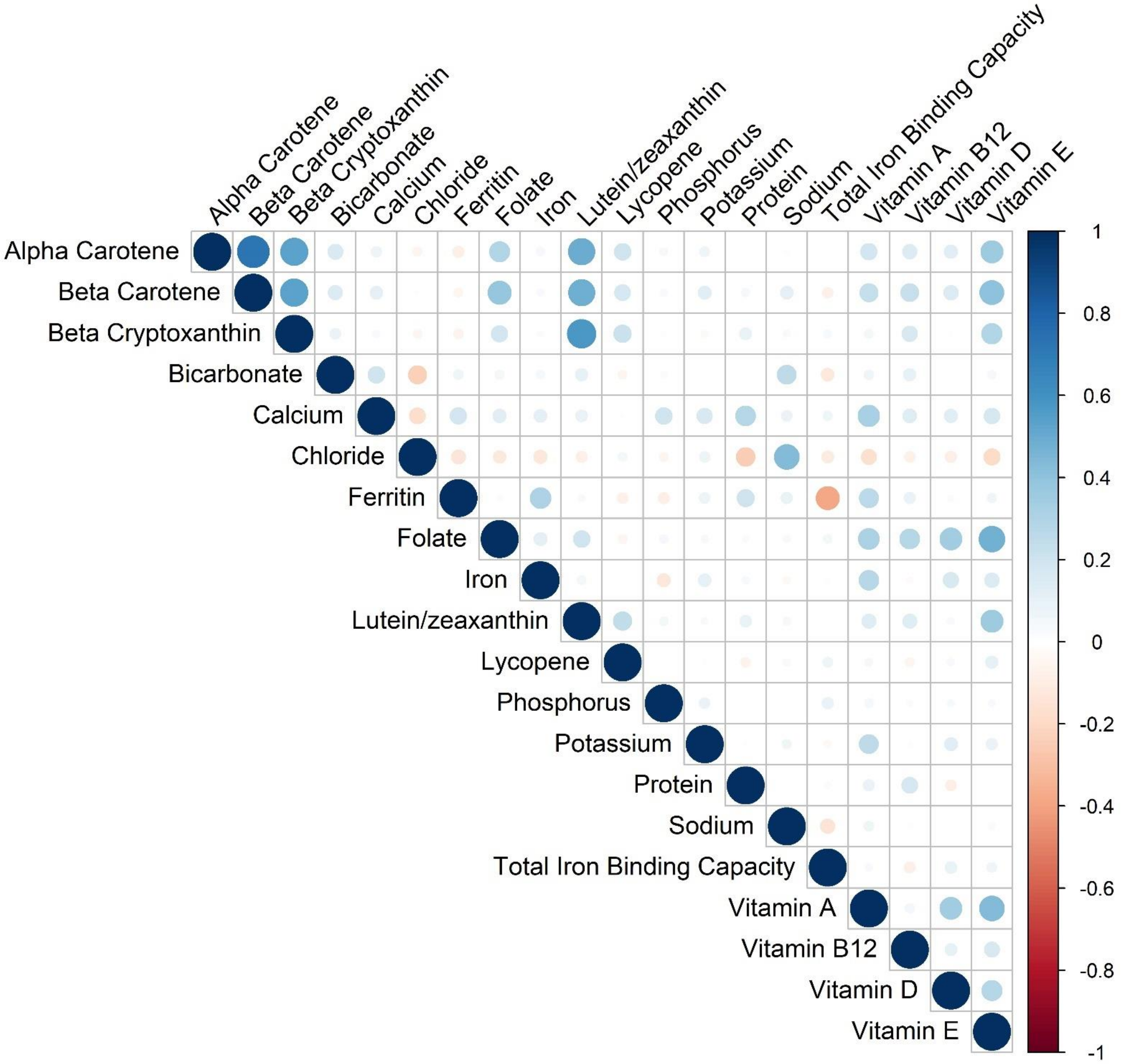

Supplemental Figure S2. Spearman correlation matrix between 20 biomarkers.

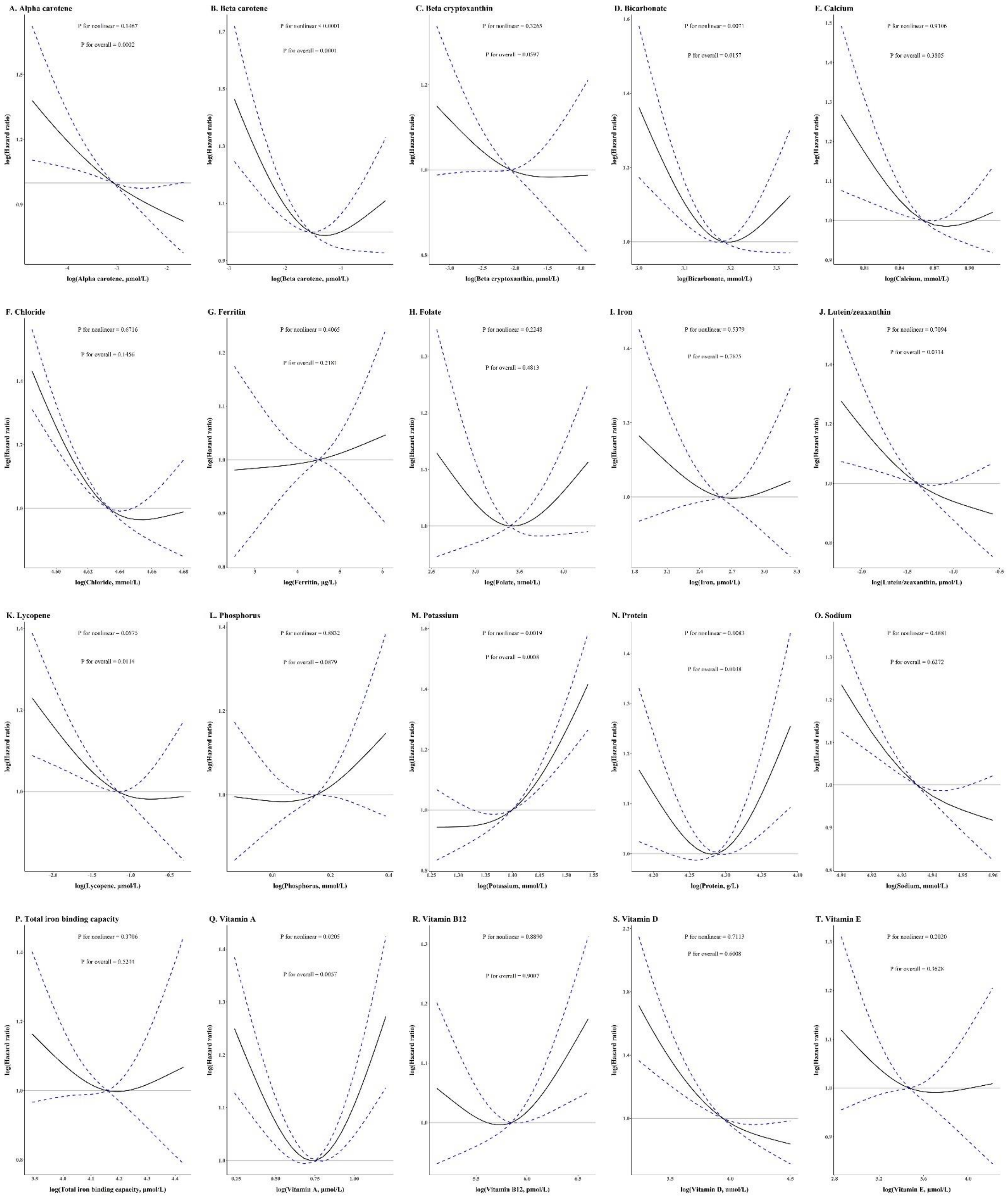

Supplemental Figure S3. Restricted cubic spline for the associations between single biomarker levels and all-cause mortality. The median concentrations were used to as reference.

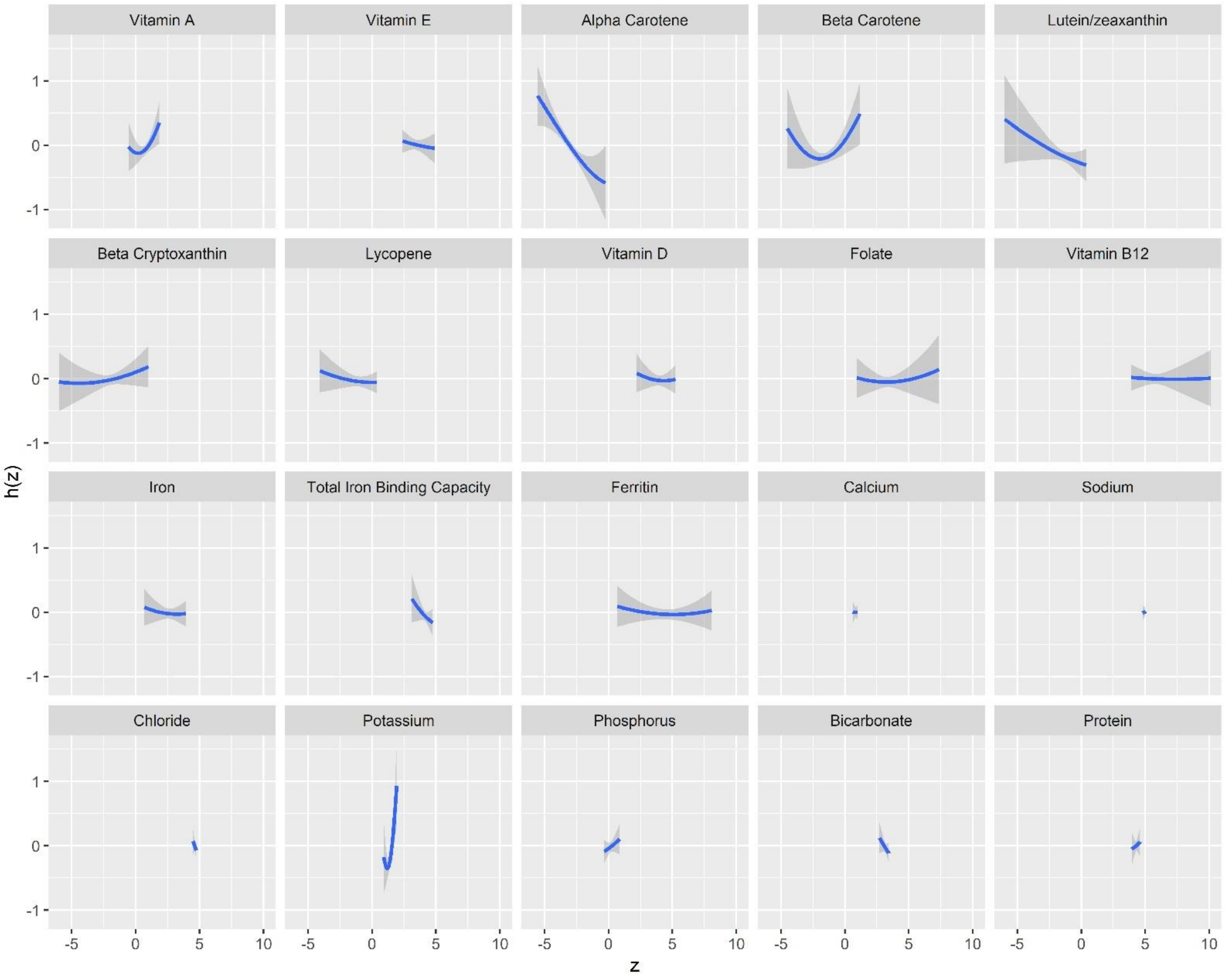

Supplemental Figure S4. Univariate concentration-response functions (95% CIs) between 20 biomarker mixtures and all-cause mortality while fixing other biomarker at their median levels. The BKMR model adjusted for age, sex, race/ethnicity, BMI, smoking status, drinking status, education, PA, annual family income, and history of chronic disease

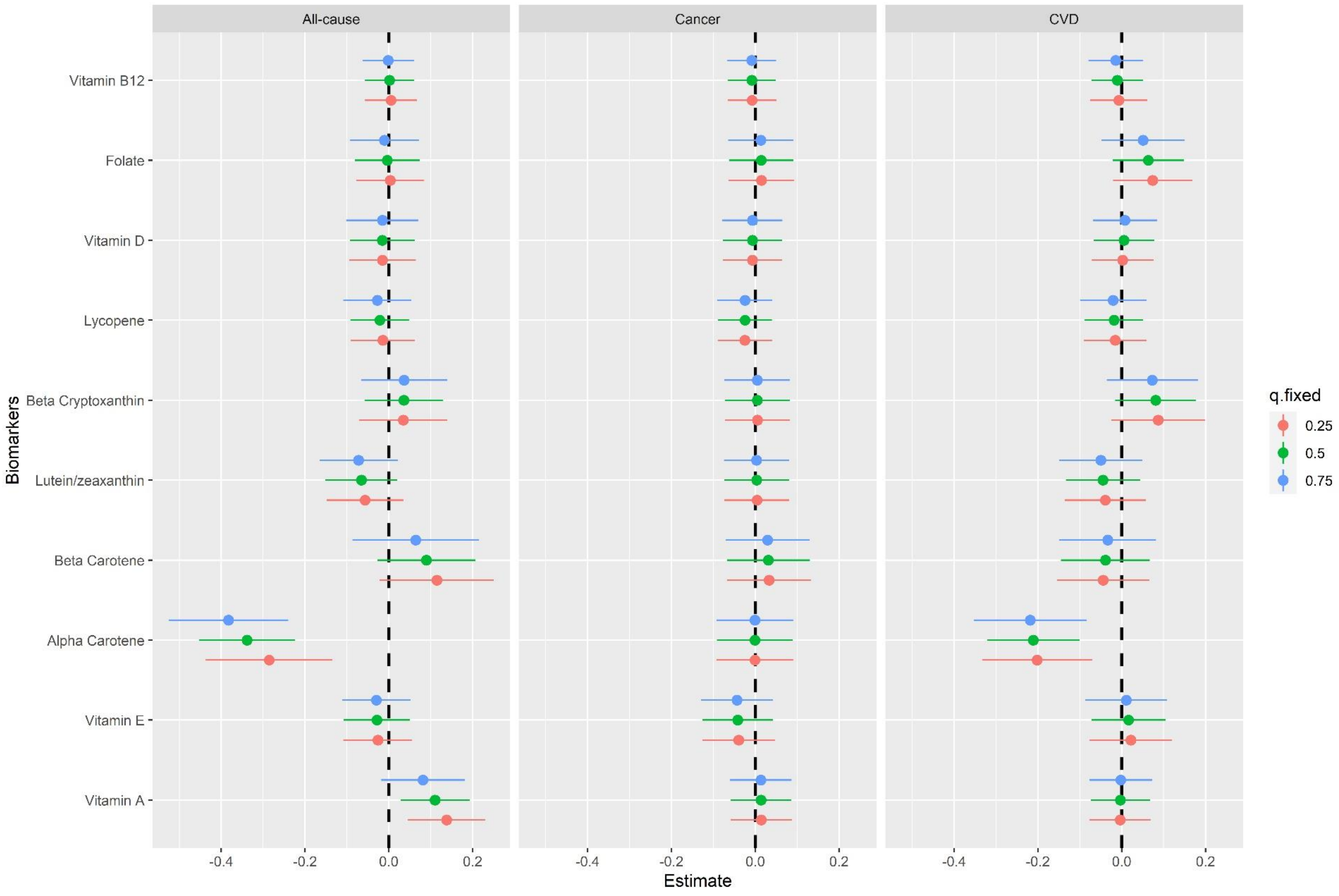

Supplemental Figure S5. Single-biomarker health effects (95% CI) in vitamin mixture, defined as the change in the response associated with a change in a particular biomarker from its 25th to its 75th percentile, where all the other biomarkers are fixed at a specific quantile. Red, green, and blue represent the 25th percentile, 50th percentile and 75th percentile, respectively.

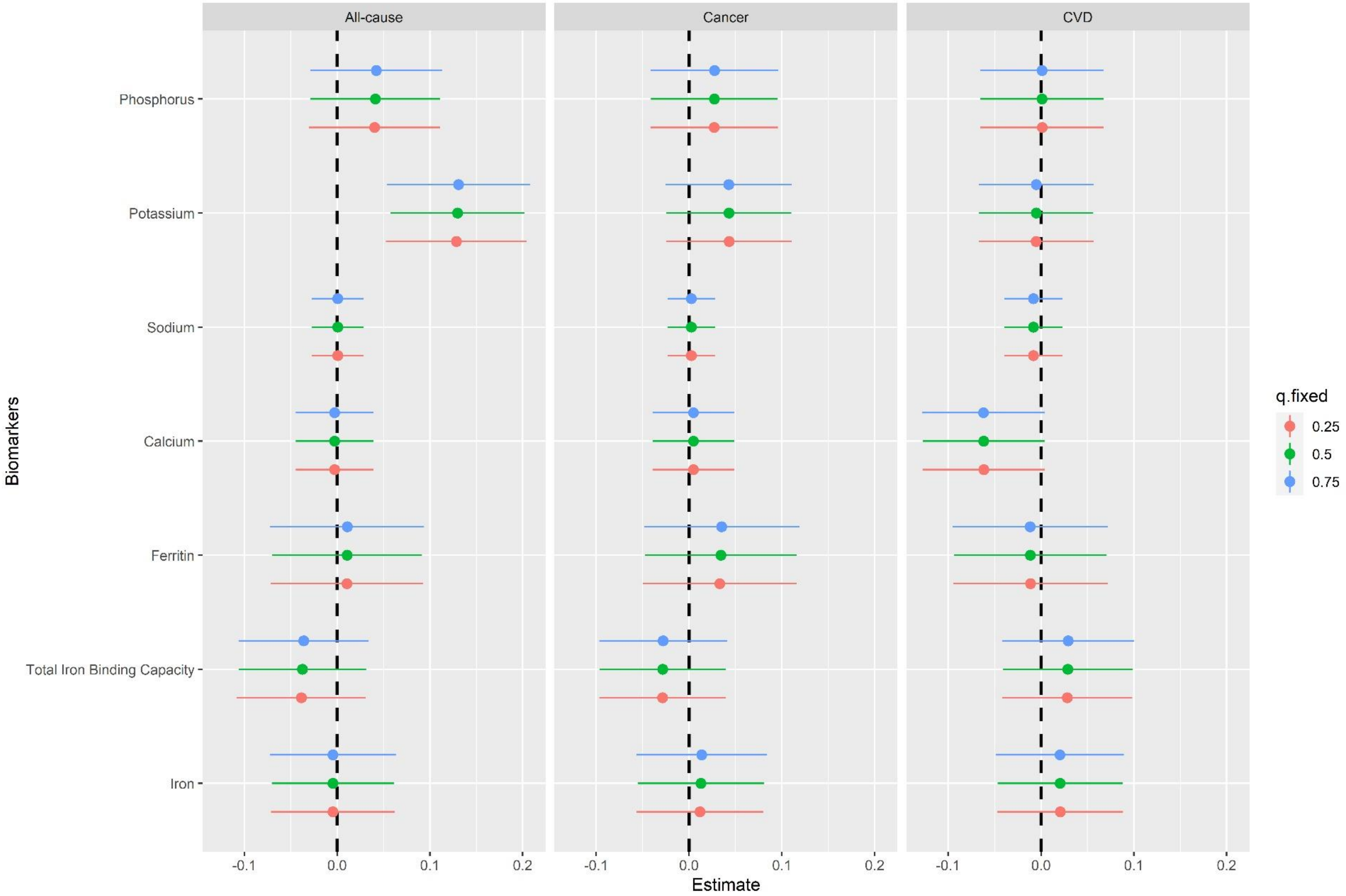

Supplemental Figure S6. Single-biomarker health effects (95% CI) in trace metal element mixture, defined as the change in the response associated with a change in a particular biomarker from its 25th to its 75th percentile, where all the other biomarkers are fixed at a specific quantile. Red, green, and blue represent the 25th percentile, 50th percentile and 75th percentile, respectively.

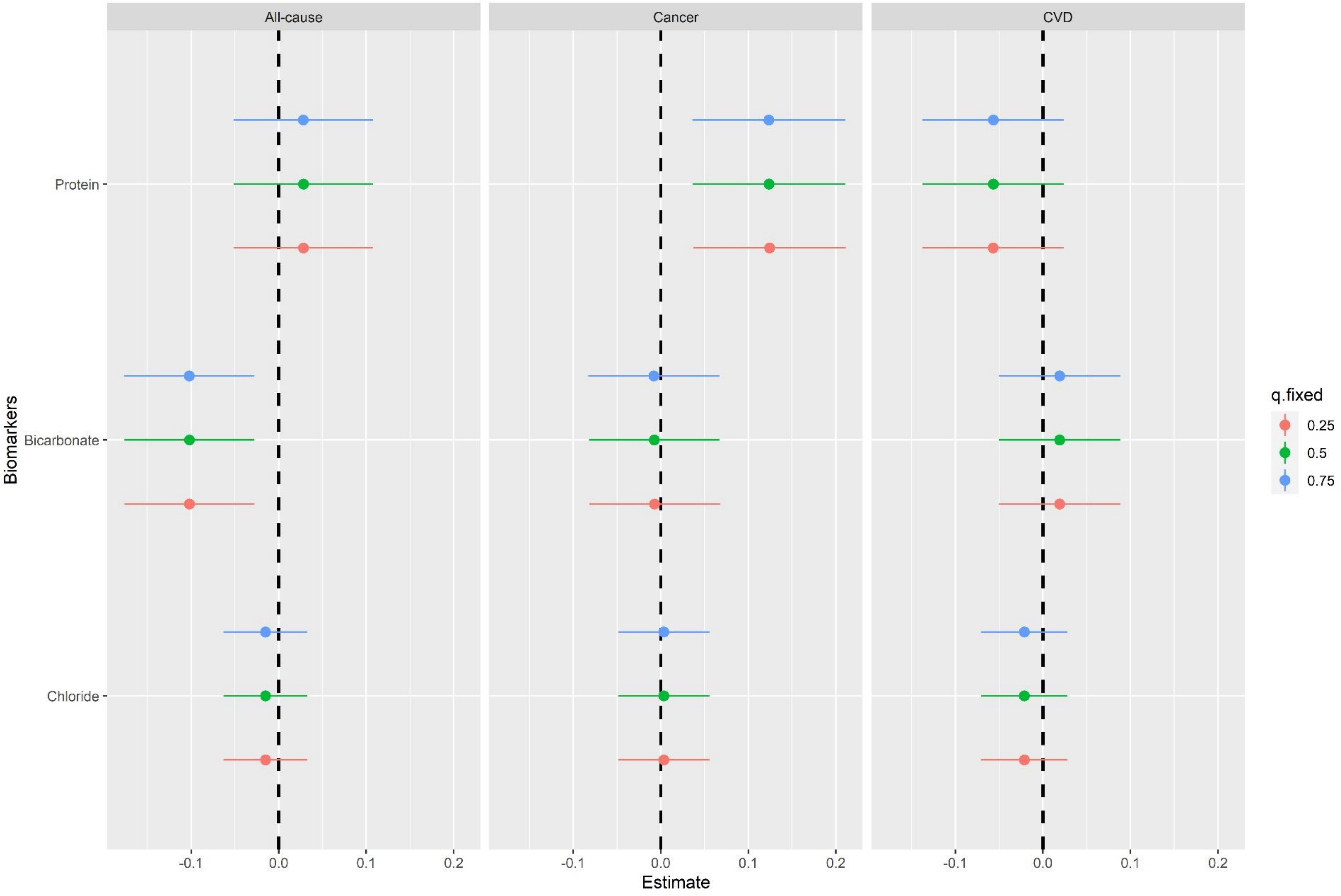

Supplemental Figure S7. Single-biomarker health effects (95% CI) in other biomarker mixture, defined as the change in the response associated with a change in a particular biomarker from its 25th to its 75th percentile, where all the other biomarkers are fixed at a specific quantile. Red, green, and blue represent the 25th percentile, 50th percentile and 75th percentile, respectively.
